# Combined oral antibiotics and intrauterine perfusion can improve in vitro fertilization and embryo transfer pregnancy outcomes in patients with chronic endometritis and repeated embryo implantation failure

**DOI:** 10.1186/s12905-023-02443-8

**Published:** 2023-06-30

**Authors:** Nana Ma, Jiaxu Li, Junlei Zhang, Yufu Jin, Jiawei Wang, Weili Qin, Fu Hang, Aiping Qin

**Affiliations:** 1 Center of Reproductive Medicine, The First Affiliated Hospital of Guangxi Medical University, Nanning, China; 2 Department of Sports Medicine, Southern University of Science And Technology Hospital, Shenzhen, China

**Keywords:** Oral antibiotics, Intrauterine perfusion, Repeated implantation failure, Chronic endometritis, Pregnancy outcomes

## Abstract

**Background:**

The aim of this retrospective study was to investigate whether oral antibiotics (doxycycline and metronidazole) combined with intrauterine perfusion (gentamicin and dexamethasone) are beneficial for patients with repeated implantation failure (RIF) and chronic endometritis (CE) to improve clinical pregnancy outcomes.

**Methods:**

Patients with RIF and CE were diagnosed using hysteroscopy and histology together. A total of 42 patients were enrolled in the study. All patients received oral antibiotics (doxycycline combined with metronidazole) and 22 patients underwent intrauterine perfusion (gentamicin combined with dexamethasone) immediately after the end of oral antibiotic therapy. Pregnancy outcomes were evaluated during the first in vitro fertilization (IVF) and embryo transfer (ET) cycle.

**Results:**

For the first D3 ET after treatment with oral antibiotics (doxycycline and metronidazole) combined with intrauterine perfusion (gentamicin and dexamethasone), higher embryo implantation rate (30.95% vs. 26.67%, *P* = 0.0308), clinical pregnancy rate (30% vs. 50%, *P* < 0.001), live birth rate (33.33% vs. 45.45%, *P* < 0.0001). No fetal malformations or ectopic pregnancies were observed.

**Conclusion:**

We report oral antibiotics (doxycycline and metronidazole) combined with intrauterine perfusion (gentamicin and dexamethasone) as a novel treatment for CE to improve the outcomes of successful pregnancy compared with those of oral antibiotics alone.

## Background

Repeated implantation failure (RIF) is defined as the inability to conceive following 3 or more consecutive embryo transfer cycles and each cycle transfer 1 ~ 2 high-quality embryos [1–3] and affects 12%–46% of patients experiencing infertility [3], which have caused great psychological distress to women [4]. RIF can result from a variety of causes, such as anomalies in the uterine cavity, endometrial non-receptivity, immunological factors, and developmental origins [3, 5]. Over recent years, an expanding body of research has shown that less receptive endometrium caused by local immune dysregulation may be the main cause of RIF [6].

Chronic endometritis (CE), which cannot be diagnosed using ultrasound and hysterosalpingography (HSG), is a chronic inflammatory condition of the endometrium without clear clinical signs. Immunohistochemistry has revealed the presence of plasma cells in the endometrial stroma. In the endometrium, CE generates an aberrant immunological milieu that results in the generation of abnormal levels of inflammatory mediators [7]. This decreases the endometrial capacity to receive embryos, which prevents implantation.

Diagnosis of CE is complicated and difficult. Currently, there are no universal diagnostic methods for endometritis. On hysteroscopy (HSC) [8], local endometrial redness and local or diffuse endometrial congestion are seen as "strawberry signs” in addition to endometrial interstitial edema, a pale and convex surface, and multiple tiny polyps. The identification of CD138 + cells by histopathology after curettage is considered a simple and objective method [9]. Among the many methods, five plasma cells per high-power field (HPF) is the most reliable method [10]. Intrauterine microbial detection can also be used as an auxiliary method, which is more complex to operate and has many factors that affect the results. In our study, we referred to hysteroscopic and histopathological findings to diagnose CE [11].

CE treatment involves administration of various antibiotics [10], and doxycycline combined with metronidazole is considered effective [12]. Doxycycline is a broad-spectrum antibiotic that is effective against both Gram-positive and Gram-negative bacteria, while metronidazole is a broad-spectrum anti-anaerobic bacteria drug that shows good effects against most bacilli. While oral antibiotics are mainly used to treat CE, improvements in pregnancy outcomes are not evident [13–15]. In a previous study [16] of patients with RIF, one group received doxycycline combined with metronidazole orally and the other group received a ciprofloxacin intrauterine infusion based on oral medication. After subsequent in vitro fertilization and embryo transfer (IVF-ET), there were no differences in embryo implantation rates, clinical pregnancy rates, or live birth rates between the two groups.

Other treatments have been investigated to achieve better results. Intrauterine administration is considered an effective method for the treatment of intrauterine diseases because of its rapid onset of action and high local drug concentrations. Antibiotic therapy in combination with platelet-rich plasma(PRP) is said to improve pregnancy outcomes in these patients, but the procedure has some limitations because it is a blood product and carries the risk of infection [17]. A recent study showed that intrauterine antibiotic infusions led to successful pregnancies in three patients with CE, for whom IVF had previously failed [15]. Oral doxycycline combined with the local administration of antibiotics can significantly improve implantation and pregnancy rates, and the effect is better than that of oral antibiotics alone [16, 18]. Another study showed that intrauterine administration of dexamethasone successfully improved endometrial receptivity. Gentamicin, the main drug used in the treatment of endometritis, is cheap and effective and has been widely used in obstetrics and gynecology [19]. Therefore, doxycycline combined with antibiotics and dexamethasone for the treatment of CE is feasible and worthy of further investigation.

In this study, we used doxycycline combined with intrauterine perfusion (gentamicin and dexamethasone) to further investigate whether this combination improves pregnancy outcomes, compared with oral administration of antibiotics alone, to find a new alternative method for the treatment of CE and to provide a new perspective for the treatment of RIF.

## Methods

### Inclusion population

We retrospectively analyzed patients who visited the reproductive centers of The First Affiliated Hospital of Guangxi Medical University between July 2019 and June 2021. All agreed and signed the informed consent form. Our screening included thyroid function and antibody testing as well as evaluation of the uterine cavity using ultrasound. During infertility evaluation, ultrasonography was performed to assess uterine anatomy and exclude those with abnormal uterine morphology.

We included women who underwent HSC and biopsy, were aged < 40 years, and had a body mass index (BMI) between 18 and 30 kg/m^2^. We excluded those with factors affecting pregnancy outcomes, such as parental chromosomal abnormalities, antiphospholipid syndrome, severe thyroid dysfunction, and autoimmune disease. All patients underwent at least three ETs with good-quality embryos (grade A), and none became pregnant. HSC was performed to clarify the cause, and a small amount of tissue was scraped for pathological examination.

### Diagnosis and histological evaluation of CE

**Fig. 1:**
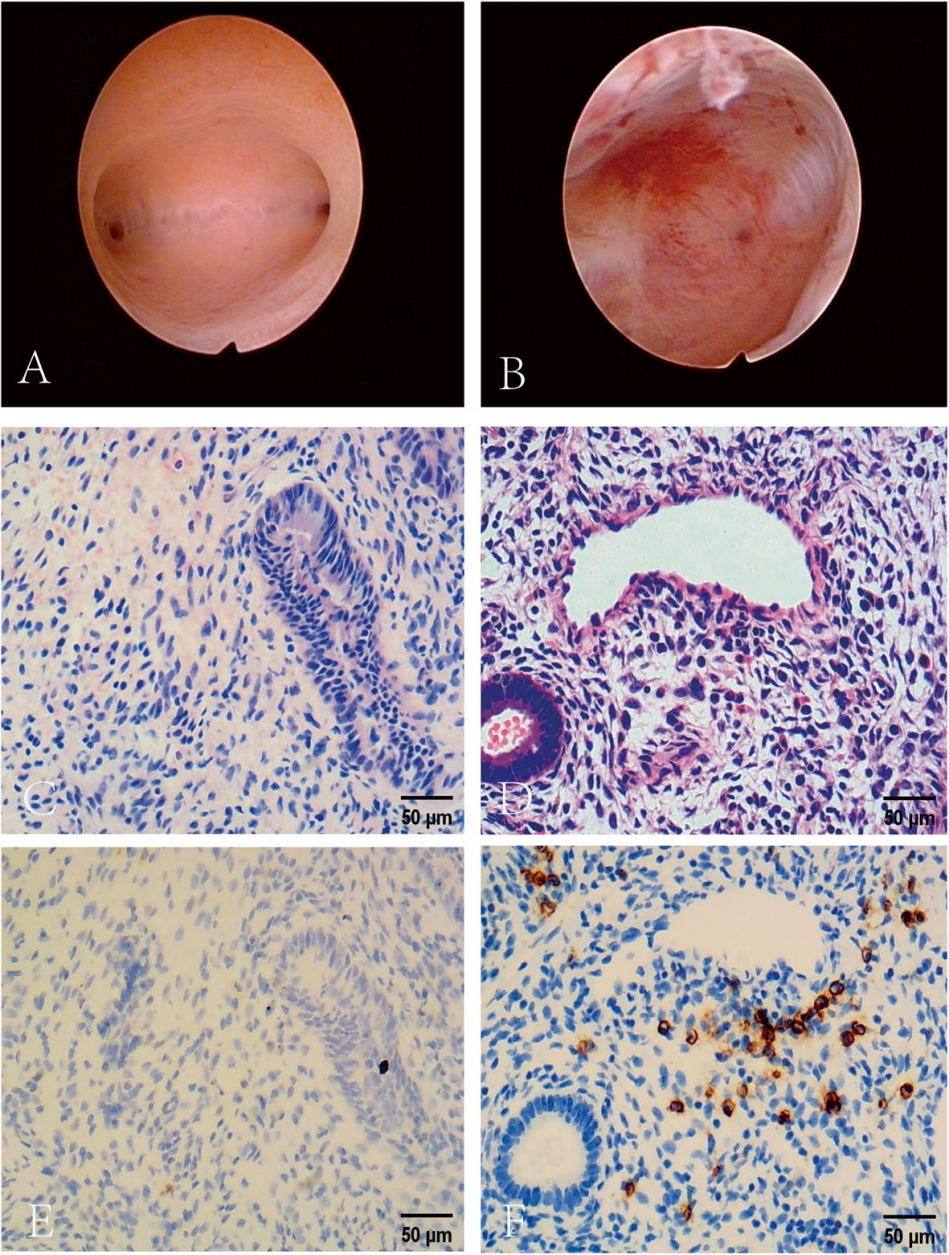
Hysteroscopy, HE staining, and immunohistochemical CD138 staining were used to compare images of patients with and without CE (× 200 magnification). Hysteroscopy (**A**. **B**), HE (**C**. **D**), and CD138 immunohistochemistry (**E**. **F**) were used to compare the normal endometrium (**A**. **C**. **E**) with that affected by chronic endometritis (**B**. **D**. **F**). CE, chronic endometritis; HE, hematoxylin–eosin; HSC, hysteroscopy

We used a cervical vaginal swab to rule out *Chlamydia trachomatis* and *Neisseria gonorrhoeae* infection before HSC. In the late follicular phase, we performed HSC and biopsy in the office without anesthetic. We photographed the uterine lumen and endometrium (Fig. 1A and B). After the intrauterine HSC inspection, we used a tiny scraping spoon to gently scrape the endometrium three times in the biopsy group. Endometrial tissue was stored in a 10% formaldehyde solution.

After paraffin embedding, serial sections with a heat treatment of 95 °C for 20 min on tissues with a thickness of 3 µm. After gradient deparaffinization and dehydration, the samples were stained with hematoxylin and eosin (HE) according to the routine protocol to distinguish the interstitial part. A dilution of 500 µL was incubated with antibodies against CD138 (Protech, Wuhan) for 1 h for simultaneous DAB coloration, and CD138 + appeared as yellow or brown-yellow particles in the interstitium. We used immunohistochemistry to diagnose CE as previously described and assessed ≥ 5 plasma cells per HPF at × 400 magnification [11] (Fig. 1 C-F).

### Therapy for CE

**Fig. 2:**
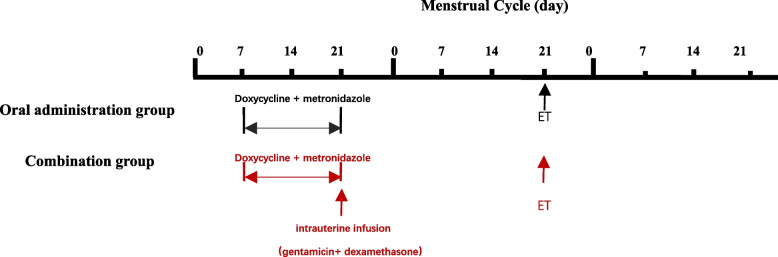
Flow of the treatment plan. Outline of the duration of treatment for each study group. During the first 14 days of treatment, all patients received oral therapy twice daily. After the oral course, the patients in the combination group received intrauterine fluid only once. All patients underwent D3 cleavage stage transplantation during the next menstrual cycle

Immunohistochemistry and HSC were used to diagnose CE in women. In cases of CE, patients received antibiotic treatment according to the French recommendations for pelvic inflammatory diseases [20, 12]. Oral antibiotics administered for CE treatment included doxycycline combined with metronidazole. Doxycycline (100 mg) was administered twice daily for 14 days. During the same period, metronidazole (400 mg) was also administered twice daily. Investigators were informed and agreed to the assigned treatment and the associated advantages and disadvantages. After oral doxycycline treatment, 22 patients were injected with a mixture of gentamicin (80 mg), dexamethasone (5 mg), and 0.9% normal saline (3 mL) into the intrauterine cavity using a sterile thin, soft tube(Fig. 2). All patients underwent re-endometrial biopsies at the end of treatment and were negative for CD138.

### Fertility treatment

Pregnancy outcomes were evaluated only for the first ET after the treatment. Embryos were usually transferred within 3 months following the end of treatment. An IVF procedure identical to the previous procedure was used. Agonist or antagonist regimens were used throughout the IVF treatment cycles, which were either natural or conventional gonadotropin-stimulated. We followed the usual protocol for endometrial preparation. In view of the fact that frozen embryo transplantation does not increase the incidence of neonatal complications and even has a better cognitive function on the nervous system [21], all patients were treated with frozen embryo transplantation after CE treatment. The cleavage stage was used to transfer all of the embryos (D3). After ET, progesterone was routinely supplemented until 12 weeks of gestation.

The following outcome indicators were observed: embryo implantation rate, clinical pregnancy rate, early abortion rate, live birth rate, and occurrence of fetal malformations.

### Statistical analysis

Comparing categorical variables, such as specific clinical traits, implantation rates, clinical pregnancy rates, live birth rates, and clinical attrition rates, was performed using chi-square testing. A t-test was used to evaluate continuous variables. SPSS 17.0 (International Business Machines Corporation, USA) was used to perform statistical analyses. At *P* < 0.05, any bidirectional *P*-value was deemed statistically significant.

## Results

### Diagnosis and treatment of patients with CE

**Fig. 3:**
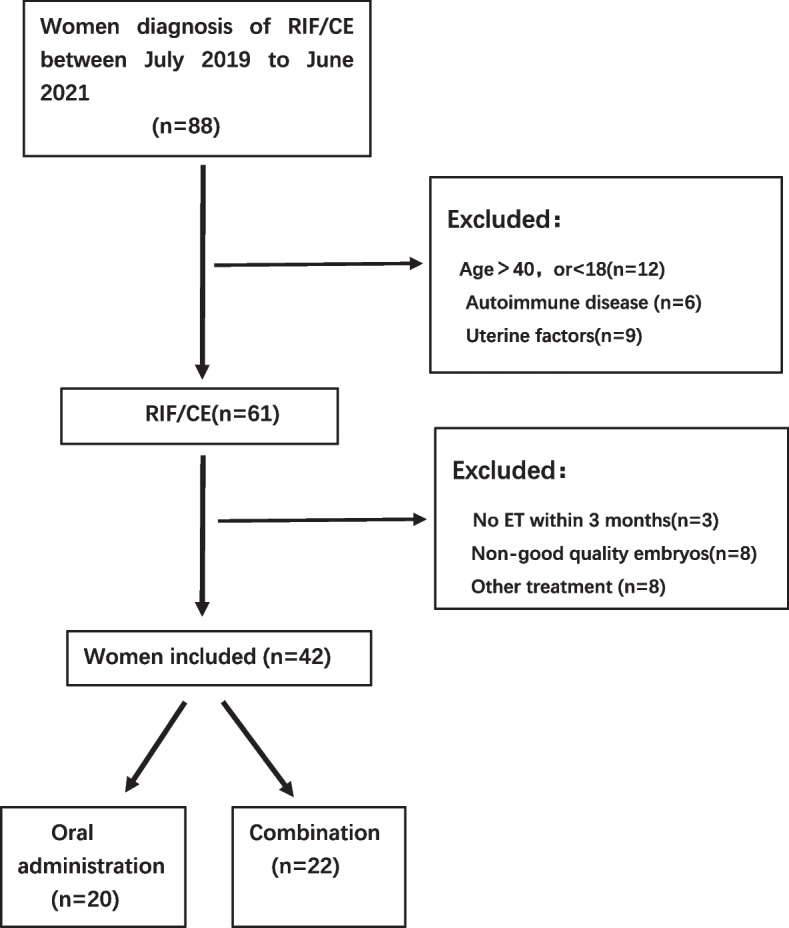
Flow chart of experimental design

A flowchart of the study is shown in Fig. 3. After excluding those who were not associated with pregnancy and those who did not undergo HSC examination or had pathological evidence of CD138 in fewer than five cells per HPF, 88 patients with a diagnosis of RIF/CE were screened between July 2019 and June 2021. All patients underwent hysteroscopy and pathology twice, qualified for inclusion and turned negative for CD138 after treatment. After the simple screening, six cases were complicated with autoimmune diseases, such as systemic lupus erythematosus, Sjogren's syndrome, and antiphospholipid syndrome, while nine were excluded due to adenomyosis, intrauterine adhesions, and uterine malformations. Additionally, 12 patients were aged either > 40 years or < 18 years. We excluded those who had not had an embryo transplant within 3 months or had no transferable dominant embryos or other treatment. A final total of 42 patients participated in this study. These patients met the following criteria: history of three failed embryo implantations, suspected CE on HSC (strawberry appearance), endometrial edema, endometrial irregularity, hyperemic areas with marked white spots (Fig. 3), and pathological diagnosis of CD138 in five or more cells per HPF. All patients received doxycycline combined with metronidazole orally. Among these, 22 patients underwent uterine perfusion with gentamicin and dexamethasone immediately after the end of oral antibiotic administration. All patients underwent IVF-ET within 3 months following the end of treatment.

### Population characteristics

The main clinical characteristics of the patients included in our study are shown in Table 1. Ovarian stimulation and ET were successfully performed in all cases using IVF-ET cycles within 3 months of treatment. We only collected results of the first ET after treatment. There were no significant differences in levels of anti-mullerian hormone (AMH), follicle-stimulating hormone (FSH), and estrogen (E2) levels or age, BMI, and infertility years between the two groups (*P* > 0.05).

**Table 1:** Clinical characteristics of women enrolled in the study

	**Oral administration group** (*n* = 20)	**Combination group** (*n* = 22)	***P* Value**
Maternal age (years) (mean ± SD)	33.05 ± 2.46	32.23 ± 3.41	0.3726
Maternal BMI (kg/m^2^)	21.06 ± 2.95	21.58 ± 2.71	0.5521
Years of infertility	5.43 ± 3.56	5.14 ± 2.9	0.7689
Cause of infertility			
Primary infertility	7	9	
Secondary infertility	10	13	
Male factor	11	17	
Tubal factor	16	18	
AMH	2.92 ± 2.59	2.90 ± 2.23	0.7756
FSH	7.07 ± 2.50	7.25 ± 3.45	0.8434
E2	39.39 ± 19.20	45.93 ± 24.90	0.8332
Endometrium thickness on ET day(mm)	9.69 ± 1.32	9.13 ± 1.73	0.8783

### Embryo implantation rate

The embryo implantation rate, which represents the transfer of gestational sacs/embryos, is an important indicator for evaluating patients with RIF. One of the main reasons for RIF is the change in endometrial receptivity, and an increase in implantation rate indicates a change in endometrial receptivity. In our study, the incidence of implantation was significantly greater in individuals who received oral antibiotics combined with intrauterine infusion than those who received oral antibiotics alone (30.95% vs. 26.67%, *P* = 0.0308, Table 2).

**Table 2:** Changes in implantation rates between the two treatments

	**Oral administration group** (*n* = 20)	**Combination group** (*n* = 22)	*P*
Number of embryos planted	30	42	
No. of gestation sac	8	13	
implantation rate	0.26667	0.309524	0.0308

### Pregnancy outcomes

The ultimate goals of all treatments are to improve pregnancy outcomes, increase live birth rates, and reduce abortion rates. We observed a higher clinical pregnancy rate (30% vs. 50%, *P* < 0.001), continuous pregnancy rate (33.33% vs. 45.45%, *P* < 0.0001), and live birth rate (33.33% vs. 45.45%, *P* < 0.0001) (Table 3). We also observed a statistically significant decrease in the rate of early pregnancy loss (6.667% vs. 4.545%, *P* = 0.0068) in the combination group compared with the oral administration group (Table 3). As for the statistics of early pregnancy loss, since there is only one case in each group, strictly speaking, the data cannot represent the difference in statistical rates, so it is necessary to expand the sample size and continue the statistics. We did not observe any ectopic pregnancies, fetal malformations, or preterm births with either treatment.

**Table 3:** IVF variables and reproductive outcomes in the first IVF-ET cycle

	**Oral administration group** (*n* = 20)	**Combination group** (*n* = 22)	*P*
Clinical pregnancy rate	6(30%)	5(50%)	< 0.0001
Early pregnancy loss rate	1(6.67%)	1(4.546%)	0.0068
Ectopic pregnancy	0	0	
Ongoing pregnancy rate	5(33.33%)	10(45.46%)	< 0.0001
Preterm birth rate	0	0	0
Live birth rate	5(33.33%)	10(45.46%)	< 0.0001
natural labor	1(6.67%)	4(18.18%)	
cesarean delivery	4(26.67%)	6(27.27%)	

## Discussion

RIF, which directly affects the pregnancy rate, is a difficult problem for reproductive doctors. In recent years, researchers have made significant efforts to study the pathogenesis and treatment of RIF with little success. Recent studies suggest that endometrial immune disorders may lead to immune cell abnormalities, involving natural killer (NK) cells, macrophages, regulatory T cells (Tregs), and other functional abnormalities, leading to maternal–fetal immune disorders and embryo implantation disorders [22]. The use of immunosuppressants, including intravenous immunoglobulin [23], intrauterine infusion of peripheral blood mononuclear cells [24], tacrolimus [25], subcutaneous administration of granulocyte colony-stimulating factor [26], and prednisone [27], have also been shown to improvement RIF and enhance the receptivity of the endometrium and improve the success rate of pregnancy. Scientists are even combining knowledge with computer science through machine learning algorithms, using artificial intelligence (AI) tools to analyze information about RIF patients and embryos to predict pregnancy outcomes [28]. In the near future, it may even be possible to use Artificial Womb Technology (AWT) to solve pregnancy problems in such patients [29].

Endometritis is an unsolved problem today. It is a significant contributor to repeated implant failures and other fertility problems. The clinical symptoms of endometritis are often mild and easily ignored. Endometritis is a complicated disease. First of all, the diagnostic methods and diagnostic criteria of endometritis are not uniform. Secondly, the treatment of endometritis is mainly empirical, the therapeutic effect is not ideal and easy to produce drug resistance. Finally, it is difficult to have a non-invasive way to check the effect of treatment after treatment.

Dexamethasone, a glucocorticoid, has powerful immunomodulatory effects. Zhang [18] administered an intrauterine infusion of dexamethasone to eight patients with RIF and found that the proportion of NK cells was downregulated in samples taken by endometrial biopsy. Seven patients successfully became pregnant after cryopreserved ET. Three patients delivered healthy babies at term without any pregnancy complications, suggesting that prednisone can downregulate the proportion of NK cells, improve endometrial receptivity, and promote embryo implantation. Huang [30] treated patients with RIF using prednisone and found that the proportion of Tregs and IL-10, which are indicators of immune tolerance, was significantly increased, whereas IL-17A, IL-23, and other proinflammatory factors were significantly decreased. After treatment, five patients with RIF successfully became pregnant and four had live births (*n* = 19). It has been suggested that prednisone can improve local immunity and place the endometrium in a sensitive phase, which is beneficial for embryo implantation.

In a study of patients with RIF, Kotaro [31] found that 33.7% of women with RIF infertility were diagnosed with CE. In a 10-year study of patients with RIF, Kotaro [32] also found that 31.4% of the women concurrently had CE and 7.8% of women with RIF had multi-drug-resistant CE, with a steady increase in multi-drug resistance (odds ratio [OR], 8.27; 95% confidence interval [CI], 2.58–26.43; *P* < 0.001). Therefore, a more effective method may be required to treat CE. Intrauterine perfusion can enable the drug to directly act more locally, quickly, and with a high concentration to reduce systemic adverse reactions. Intrauterine perfusion is a commonly used treatment in obstetrics and gynecology, and it is the first-line treatment for endometritis during labor [32], with high levels of safety and effectiveness. In animal experiments, intrauterine infusion of gentamicin and dexamethasone in buffaloes with endometritis was shown to significantly increase the number of pregnancies [32]. Zhang [18] treated patients with RIF/CE using an intrauterine infusion of dexamethasone combined with gentamicin/clindamycin, with a 60% cure rate of CE, indicating that gentamicin has good therapeutic effect on CE. The clinical pregnancy and live birth rates of patients with treated RIF/CE were increased after cryo-ET, not only because of the therapeutic effect of gentamicin on CE but also because dexamethasone treatment can increase endometrial embryo acceptance and improve pregnancy outcomes. Therefore, we believe that oral doxycycline and metronidazole combined with an intrauterine infusion of gentamicin and dexamethasone may achieve an improved therapeutic effect.

In our study, we retrospectively analyzed patients with RIF/CE between July 2019 and June 2021. After oral doxycycline and metronidazole administration, patients were administered an intrauterine infusion of gentamicin and dexamethasone, and pregnancy outcomes were observed after ET. Our findings support that oral antibiotics combined with an intrauterine infusion of antibiotics have a better effect on pregnancy outcomes than do oral antibiotics alone and can improve implantation rate, clinical pregnancy rate, and live birth rate (all *P* < 0.05).

Our inclusion requirements are quite tight, which is why we only have a tiny number of examples. Another important factor was that we did not include these patients who did not have their CD138 levels checked again or who did not have two standardized hysteroscopies. Because the patients we included were retransplanted after RIF, and some of the patients had no optimal embryo, we had no way to evaluate the effectiveness of the method. Further decreasing the number of instances we considered was the fact that some patients were also not included in other therapies.

To assess the effect of antibiotic treatment on CE and the integrity of the study, we initially planned to include women with RIF/CE and either placebo or no treatment in the control group. However, we would not find patients in the hospital who were diagnosed with RIF/CE and did not receive any treatment, it's not ethical. We also excluded patients who were treated with other regiments. Given the large number of reports on the efficacy of oral antibiotics (doxycycline and metronidazole) in the treatment of CE, we compared whether oral antibiotics combined with an intrauterine infusion of antibiotics were more effective than oral antibiotics alone.

## Conclusion

In conclusion, our results show that the use of oral antibiotics (doxycycline and metronidazole) combined with intrauterine perfusion (gentamicin and dexamethasone) are beneficial for patients with RIF/CE. When D3 cleavage were transplanted, the combination group showed higher implantation, clinical pregnancy, and live birth rates. These drugs are widely used in the fields of obstetrics and gynecology and have no teratogenic effect. We plan to conduct further animal experiments to elucidate the specific molecular mechanisms.

## Data Availability

All the data and materials were obtained from the First Affiliated Hospital of Guangxi Medical University, and the data were real and reliable. The datasets used and/or analysed during the current study available from the corresponding author on reasonable request.
